# The gap between attitudes toward life, death, and bereavement and the actions of local health care systems: The case of Yakushima, a world natural heritage site in Japan

**DOI:** 10.7189/jogh.12.03074

**Published:** 2022-11-12

**Authors:** Yurie Kobashi, Makoto Yoshida, Tomohiko Sugishita

**Affiliations:** 1 Department of Radiation Health Management, Fukushima Medical University School of Medicine, Fukushima City, Fukushima, Japan; 2 Faculty of Medicine, Teikyo University School of Medicine, Itabashi, Tokyo, Japan; 3 Yakushima Onoaida Clinic, Yakushima Town, Kagoshima, Japan; 4 Department of Public Health, Tokyo Women’s Medical University, Shinjuku, Tokyo, Japan; Photo: The aerial view of Yakushima and Onoaida Clinic (the building in the foreground is Yakushima Onoaida Clinic). Source: from the authors’ own collection, used with permission.

Attitudes toward death and bereavement must be recognized as not only usual personal events, but also valuable and promising learning opportunities [1], and beliefs and attitudes toward life and death are important factors when considering the value of death. The era of coronavirus disease 2019 (COVID-19) pandemic has provided people with the opportunity to deliberate about death, dying, and bereavement in the social context [2]. In the 14th century, the bubonic plague pandemic in Europe popularized the artistic concept of “memento mori,” which also influenced people’s perspectives on death and dying. Memento mori, which encourages people to remember that they must die, might construct the foundation of the view of death among the western population. Subsequently, thanatology, which considers death more positively and promotes living one’s end-of-life with a sense of possibility and potential, was developed [3,4]. Of the five principles set out by the Lancet Commission on the Value of Death, three are related to beliefs and attitudes toward life and death: dying is understood to be a relational and spiritual process rather than simply a physiological event; conversations and stories about everyday death and grief become common; and death is recognized as having value [1]. These principles are an essential foundation for considering what constitutes a “good death.”

Several studies have been conducted to gain a better understanding of what constitutes a good death. One study among patients with terminal disease reported that the core elements of a good death included the control of pain and symptoms, clear decision-making, being taken and perceived as a person, and having the ability to give something to other people [5]. Another study reported that the presence of family members and not being a burden were core elements of a good death, and that the definition of a good death differed among patients, their families, and persons with different occupations [6]. Hence, the definition of a good death is individual and unique according to cultural and religious contexts, personal experiences associated with death and bereavement, as well as beliefs and attitudes toward life and death [7]. Achieving a good death is associated with personal fulfillment in society and the availability of health care service options for end-of-life treatment and terminal care [5,8,9]. However, few studies have examined the health care system for patients with terminal disease from the point of view of beliefs and attitudes toward life and death.

Yakushima is an island in Kagoshima Prefecture, located in the south of Japan. It has an area of 540.5 km^2^ and a population of 11 858 (as of 2020) [10]. The island’s population is rapidly aging, with 36.4% of the present population aged 65 years and older. 90% of the island is covered in primeval forests; approximately 20% of this land area was designated a UNESCO World Heritage site in 1993.

Yakushima is a typical local community in Japan and has rich social and cultural norms in terms of beliefs and attitudes toward life and death. Regarding the Lancet Commission, which stated that dying is understood as a relational and spiritual process rather than simply a physiological event, the cultural viewpoints of residents of Yakushima are influenced by a sense of awe toward nature. A recent book on the history of Yakushima described the residents of Yakushima as living with a sense of the awe toward “something beyond this world“, such as legendary supernatural creatures known as *yokai* and *mononoke* [11]. The residents of Yakushima recognize that they cannot control nature, life, or death, and seem to accept the process of dying as natural. They sometimes forego receiving substantial medical treatment, as they believe it would delay a natural death. In relation to the second principle outlined by the Lancet Commission on the Value of Death that “conversations and stories about everyday death, dying, and grief become common” [1], most middle-aged and older generations habitually visit the ancestral graves of their parents, relatives, friends, and neighbors daily, making conversations with the deceased natural and commonplace. Therefore, many residents of Yakushima might be considered to exemplify the attitudes toward life and death that were determined to be desirable by the Lancet commission. Nevertheless, many challenges remain regarding the opportunity to receive optimal terminal care and support for attaining a “good death” under the social and cultural contexts faced by each individual.

First, hospital-based health care in the terminal stage when facing death and dying might not match local residents’ beliefs and attitudes toward life and death. An equipped general hospital was built in Yakushima in 1997 and provides vital health care to date. However, the baseline assumption regarding the place where the residents preferred to stay during their last days shifted from their home to a general hospital; it is difficult for residents to choose the terminal days at home nowadays. A part of the residents prefers to eat fresh food, mainly obtained by fishing or from farms; they value a natural lifestyle and thus prefer to spend their last days naturally at home. In these situations, the terminal care against natural end of life was not suitable. Therefore, the significance of achieving a good death in the local context might need to be reconsidered. Increasing the number of choices for terminal care is essential to achieving a good death in the family and communal context (which involves such things as their placing a value on being in nature). Additionally, medical staff and administrative officers may need to learn more about residents’ beliefs and attitudes toward life and death, and gathering this information is urgently needed to establish an ideal end-of-life health care service based on the communal mindset.

Second, there is a lack of medical and professional resources in remote and rural areas, such as Yakushima Island. Yakushima has only one hospital, seven clinics, and eight long-term care facilities, and there is an inadequate number of medical staff, particularly those who provide long-term care. Moreover, the distances between residents’ homes and the medical facilities make it difficult for individuals to receive standard medical care. The cooperation and expertise needed to provide inclusive health care support across several sectors is a vital element of good end-of-life care [9]. Those who recognize dying as a valuable spiritual process might experience spiritual pain if they do not have an ideal end-of-life. It is therefore necessary to provide a variety of end-of-life health care options, including home-based health care, corresponding to a variety of needs.

Third, the residents of Yakushima cannot typically receive support from family members easily. Most local young residents leave Yakushima, and the majority of them do not return. Therefore, many households include only aging residents. Also, island residents form families with people outside of the island through marriage and other forms of relationships, and their beliefs regarding what constitutes a good death is often not acknowledged by all family members and relatives. Moreover, based on their close relationships with their families and local communities, many residents consider the communal happiness of their families more essential than their own individual happiness. Therefore, when considering a good death, it is vital to respect the beliefs and opinions of not only the patient, but also the family members. Previous studies have reported that a good relationship with their family was the most essential factor for a good death for patients [6]. It might be vital to educate the family and community on how family members and medical staff can work together to support a good death for the patient. To avoid cases in which the patient’s beliefs are ignored and they experience pain from not having ideal terminal health care, even in the case of disagreements among family members, family members and the community need to share the patient’s beliefs and values with regard to death; and key persons need to foster their decision-making skills as well. Advanced care planning with written consent from the patient as witnessed by family members could be one of the solutions.

The gap between ideal end-of-life health care among patients and the actual options of health care might cause great spiritual pain. Appropriate health care systems that can provide comprehensive terminal care through cross-sectoral collaborations between medical care, nursing care, and welfare, in conjunction with the patient’s individual beliefs regarding life and death, are needed. Furthermore, similar to how the bubonic plague affected views of death, dying, and bereavement in the 14th century, the recent COVID-19 pandemic provided people with an opportunity to reveal a limitation of end of life health care systems and reconsider their beliefs and attitudes toward death, dying, and bereavement [2]. Additionally, restrictions on hospital visits for patients with COVID-19 have also stressed an emergent need for actions towards attaining a good death [12]. These issues cannot be solved if the cultural and social contexts are ignored; therefore, strategies from the local level about issues between end-of-life health care systems and individual beliefs toward life and death are vital.
